# Optimizing Ti/TiN Multilayers for UV, Optical and Near-IR Microwave Kinetic Inductance Detectors

**DOI:** 10.1007/s10909-024-03121-1

**Published:** 2024-05-15

**Authors:** Gerhard Ulbricht, Mario De Lucia, Jack Piercy, Oisín Creaner, Colm Bracken, Cáthal McAleer, Tom Ray

**Affiliations:** 1 Dublin Institute for Advanced Studies, 31 Fitzwilliam Place, Dublin 02, Ireland; 2 School of Physics, Trinity College Dublin, College Green, Dublin 02, Ireland; 3 Department of Experimental Physics, Maynooth University, Maynooth, Ireland; 4 School of Physical Sciences, Dublin City University, Glasnevin Campus, Dublin 9, Ireland

**Keywords:** Kinetic inductance detectors, UVOIR MKIDs, Detector fabrication

## Abstract

Microwave Kinetic Inductance Detectors (MKIDs) combine significant advantages for photon detection like single photon counting, single pixel energy resolution, vanishing dark counts and µs time resolution with a simple design and the feasibility to scale up into the megapixel range. But high quality MKID fabrication remains challenging as established superconductors tend to either have intrinsic disadvantages, are challenging to deposit or require very low operating temperatures. As alternating stacks of thin Ti and TiN films have shown very impressive results for far-IR and sub-mm MKIDs, they promise significant improvements for UV, visible to near-IR MKIDs as well, especially as they are comparably easy to fabricate and control. In this paper, we present our ongoing project to adapt proximity coupled superconducting films for photon counting MKIDs. Some of the main advantages of Ti/TiN multilayers are their good control of critical temperature (*T*_c_) and their great homogeneity of *T*_c_ even over large wafers, promising improved pixel yield especially for large arrays. We demonstrate the effect different temperatures during fabrication have on the detector performance and discuss excess phase noise observed caused by surface oxidization of exposed Si. Our first prototypes achieved photon energy resolving powers of up to 3.1 but turned out to be much too insensitive. As the work presented is still in progress, we also discuss further improvements planned for the near future.

Microwave Kinetic Inductance Detectors (MKIDs) [1, 2] are photon detectors based on superconducting resonator circuits. Especially in the UV, visible to near-IR (UVOIR) range, their unique combination of important capabilities like single photon sensitivity, single pixel wavelength resolution, µs timing accuracy, lack of dark counts and multiplexibility into the megapixel range make them a very attractive detector option for many applications in modern scientific instrumentation [3]. MKIDs are not only able to detect single UVOIR photons but can also determine their individual wavelength with a demonstrated [4] resolving power *λ*/Δ*λ* of up to 52 without requiring any refractive optics. It is this single pixel, single photon wavelength resolution that is their most important advantage compared to older, more established detector principles like for example CCD or CMOS arrays.

But in order to allow MKIDs to measure the individual wavelength of every detected photon, several challenging constrains arise for both detector design and fabrication: High photon energy resolving power requires, among others, the superconducting resonator in MKID pixels to have high internal quality factors *Q*_i_ and that the used superconducting film is as homogeneous as possible. This significant demand on *Q*_i_ for example requires highly optimized nanofabrication steps and all fabrication to be performed in the cleanest possible environment. Furthermore, MKIDs are frequency-domain multiplexed (FDM). In order to achieve large detector arrays with up to 2000 MKID pixels per microwave feedline [5] without losing too many pixels to frequency overlaps, FDM requires the resonant frequency of individual MKIDs to be controlled to a degree of better than 99.99%. These difficult to achieve fabrication conditions can make MKID research and development challenging as the demanding and dedicated cleanroom fabrication infrastructure is only available in rare cases. Most university nanofabrication facilities have issues achieving the required particle counts or deposition system stabilities, often resulting in reduced detector capabilities or less then optimal pixel yield.

These high requirements for the precise control of the superconducting film’s characteristics result in a limited selection of suitable superconducting materials for UVOIR MKIDs. PtSi [6], Al [4], sub-stoichiometric TiN_x_ [7, 8] and ß-phase Ta [9] have for example been demonstrated with their respective advantages and disadvantages. Here, we report on our efforts to demonstrate proximity effect-based TiN/Ti/TiN triple layers as further alternative for UVOIR MKIDs. TiN/Ti/TiN multilayers are attractive for MKIDs for several reasons, key among them that they can be deposited and structured by commercial nanofabrication foundries and therefore allow to compensate for limitations of available fabrication infrastructure. TiN/Ti/TiN multilayers enable us to collaborate with the Tyndall National Institute in Cork, a semi-commercial foundry that provides thin film deposition, lithography and dry-etching services. Apart from their comparably easy deposition, TiN/Ti/TiN multilayers also offer full control of their critical temperature *T*_c_ by utilizing the proximity effect [10, 11], exhibit very high *T*_c_ homogeneity over a wafer and have been demonstrated to work well for far-IR and sub-mm MKIDs (see for example [12]). But as detector design and fabrication challenges differ severely between UVOIR MKIDs and MKIDs optimized for longer wavelengths, significant effort is required to go from sub-mm TiN/Ti/TiN MKIDs to optical ones. Unfortunately, it is also not possible to just copy the fabrication details demonstrated for example by NIST in [10] for their high quality multilayer films as thin film deposition characteristics vary significantly between different deposition tools. The triple-layer approach is preferable over TiN/Ti double layers mainly as having the proximity effect on both sides allows to use thicker Ti layers and therefore to achieve better control over low-*T*_c_ films.

We are working in close collaboration with our colleagues at Tyndall National Institute to further optimize the results we get from multilayer MKIDs. We use a TiN/Ti/TiN triple-layer structure as TiN at the outside reduces reflection of UVOIR photons and protects the central Ti layer from oxidization. *T*_c_ in a TiN/Ti/TiN triple-layer is dictated by its three film thicknesses: As long as both the Ti layer (bulk *T*_c_ expected to be 400 mK) as well as the stoichiometric TiN layers (*T*_c_ around 4.5 K [13]) are thin enough, the proximity effect results in a common *T*_c_ of the multilayer that will be near to the value averaged over the films. Figure 1 shows the control of *T*_c_ with film thickness we achieved. It also demonstrates that more details have to be taken into consideration, as will be discussed below.

**Fig. 1:**
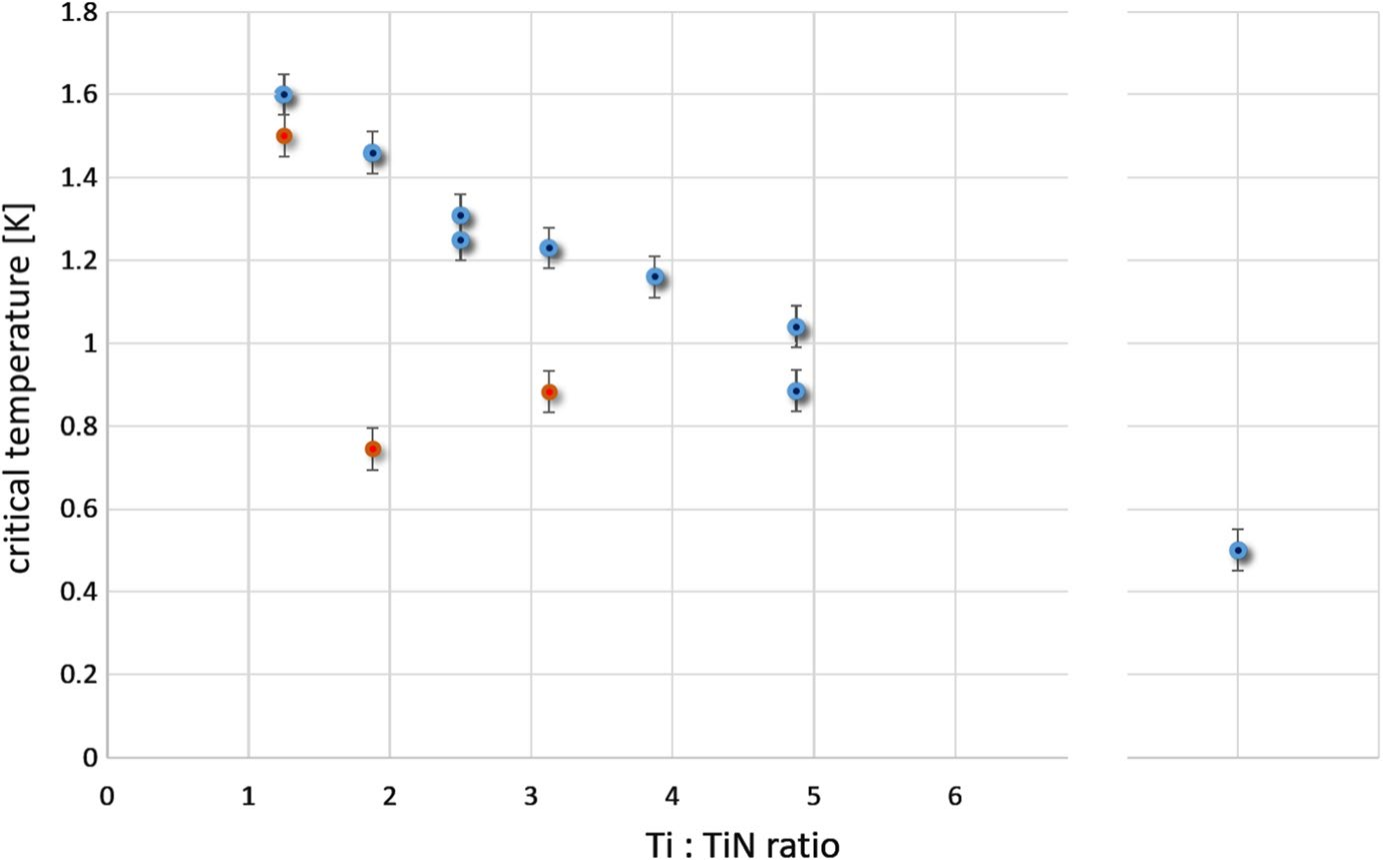
Dependency of critical temperature for 12 different TiN/Ti/TiN layers on the Ti:TiN thickness ratio in the film. For the *blue circles,* the films have been measured just after deposition. The point at the right represents a single Ti layer. The *red circles* represent *T*_c_ values after lithography and dry-etching has been performed on the wafer. This involved additional steps at elevated temperatures (not identical for the three samples) shifting *T*_c_—for details please see text. (Colour figure online.)

The red data points represent samples after lithography and etching and will be discussed further below. The blue data points in Fig. 1 represent TiN/Ti/TiN films that have been sputter deposited on Si substrates, with *T*_c_ measurements performed directly afterwards without any further fabrication steps. The TiN thickness of both layers has been kept unchanged at 4 nm for all samples, only the Ti layer has been varied between 10 and 39 nm. Layer thickness is controlled by precise control of sputter times and checked for a few samples with scanning electron microscopy. The point at the far right in Fig. 1 shows our measured *T*_c_ of 500 ± 10 mK for just a Ti layer which compares well to the expected bulk value. For a single stoichiometric TiN layer, we measure a *T*_c_ of 4.17 K. This does not completely reach the reported values in [13], indicating that our TiN layers still lack a small amount of nitrogen, but is sufficiently near to the literature value that we did not try to further increase it. This TiN recipe has been used for all TiN layers of the presented TiN/Ti/TiN films. The error bars in Fig. 1 represent our measurement inaccuracy when determining T_c_. In order to check for reproducibility, we fabricated and measured several nominally identical samples a few months apart: For one pair, we used 4 nm/39 nm/4 nm of TiN/Ti/TiN, respectively (for a Ti:TiN ratio of 4.9) and got critical temperatures of 885 ± 10 mK and 1040 mK ± 10 mK. This indicates that the deposition system our colleagues at Tyndall are using has some drift over a few months that we have to take into consideration. Otherwise, the *T*_c_ control we achieve is sufficiently precise for our purpose.

In order to achieve good resolving power in UVOIR MKIDs, it is important to use a superconductor with the lowest *T*_c_ the used cryogenic system can support. In our case that means we are targeting a *T*_c_ of around 800 mK. This forces us to use very thin TiN layers—4 nm is at the edge of what can be sputter deposited with sufficient reproducibility. This is likely to contribute to the slight drift/reproducibility issue we see. The largest Ti layer thickness we have tested so far is 39 nm. At this film thickness the proximity effect risks to no longer influence the complete film. We do not see any signs of the proximity effect not to cover the whole Ti layer yet, but as we have TiN on both sides the effective proximity thickness is half the Ti layer thickness anyways. We do observe low internal quality factors (*Q*_i_) in the 4 nm/39 nm/4 nm TiN/Ti/TiN sample (see Table 1) though. This could indicate that the inner part of the Ti layer has an effectively lower *T*_c_, but more experiments are needed to make this statement more reliable.

**Table 1:** Measured *T*_c_ and internal quality factors (*Q*_i_) for different Ti:TiN ratios

Sample name	A	B	C	D	E	F
Ti:TiN ratio	1.25	1.25	1.9	1.9	3.1	4.9
*Q*_i_	160,000	40,000	160,000	5000	80,000	19,000
*T*_c_	1.55 K	1.50 K	1.46 K	0.77 K	0.88 K	0.89 K

The shown *Q*_i_ values are averaged over several resonators but can vary widely, especially for lower quality films. Measuring *Q*_i_ requires a resonator; therefore, all these samples have undergone lithography and dry etching

When testing for *T*_c_ homogeneity, we are measuring the same critical temperature within our ± 10 mK measurement inaccuracy over a full 4″ wafer. Inhomogeneities in *T*_c_ can both contribute to bad pixel yield as well as reduced wavelength resolving power in UVOIR MKIDs. In most materials, a change in *T*_c_ is accompanied by a change in the kinetic inductance value of the film and would therefore shift the resonant frequencies of MKID resonators in an uncontrolled way, leading to pixels with too similar resonant frequencies that can’t be distinguished in the FDM readout anymore and have to be discarded. Furthermore, microscopic variations in *T*_c_ also need to be avoided as they represent changes in the superconducting band gap—the amount of broken Cooper pairs per photon could get location dependent if T_c_ is not constant everywhere, limiting the MKID’s capability to measure the photon’s energy. Highly uniform TiN/Ti/TiN multilayers thus promise to significantly increase the pixel yield of large UVOIR MKID arrays and could possibly also contribute to further increasing their resolving power.

The internal quality factors of TiN/Ti/TiN layers with low *T*_c_ we have fabricated so far are unfortunately all below 20,000, see Table 1. For a lower Ti:TiN ratio (corresponding to a higher *T*_c_), we get better *Q*_i_ values of around 160,000, in single resonators above 200,000, as shown in Fig. 2. But for lower *T*_c_ samples, the *Q*_i_ we observe drops. We do not have enough samples analysed yet though (work is ongoing), and two additional effects are influencing the values shown in Table 1: Sample D is a so far unexplained outlier—we assume some mishap during fabrication and only include it for completeness. More important is the second effect: We see significant changes in both *T*_c_ and *Q*_i_ depending on the temperatures the sample has experienced during fabrication. As shown with the red points in Fig. 1, we get consistently higher T_c_ values if we analyse films that have not been structured by lithography compared to identical wafers (they have been deposited in the same deposition run) with resonator structures etched into them. We attribute that behaviour to the extended temperatures used during sample fabrication, in our case resist baking at 150, 90 and 120 °C during lithography and resist removal in an O_2_ + N_2_ + NH_3_ plasma at above 200 °C. We assume that especially during the high-temperature resist removal step, Ti starts to diffuse into the TiN layers, changing their stoichiometry, reducing their *T*_c_ and thus reducing the film’s overall *T*_c_. First preliminary tests of this hypothesis by changing the resist removal step to room temperature show less reduction of *T*_c_ (samples B and E) but more detailed studies and especially controlled annealing experiments are required to make a reliable statement. The same unintended annealing during lithography also seems to influence *Q*_i_. By reducing the temperature during resist removal we also reduced *Q*_i_ significantly—this could be partly caused by the now lower *T*_c_ as we do observe lower *Q*_i_ for lower *T*_c_ values, and we are working on compensating for the *T*_c_ drift during lithography by adapting the Ti layer thickness. More detailed experiments on the annealing of TiN/Ti multilayers are ongoing and we hope to be able to provide better data in the near future.

**Fig. 2:**
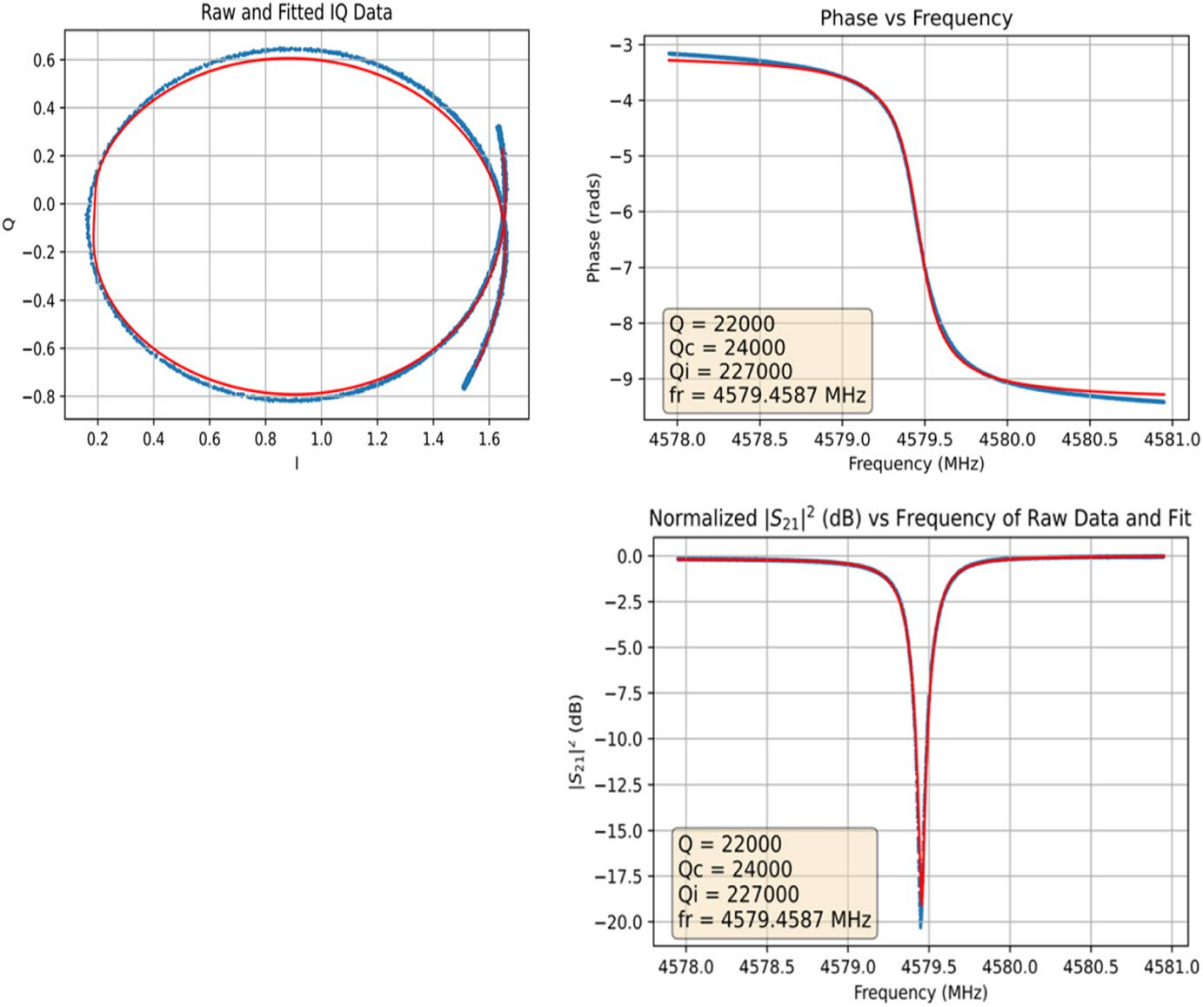
Frequency sweep in the IQ plane, S21 feedline transmission and phase versus resonator loop centre for one of the best MKIDs of sample group C (colour figure online). The frequency sweep also shows parts of the cable-delay loop as the cable phase offset has not been removed from the data

Stripping remaining photo resist after lithography at elevated temperatures in an O_2_ + N_2_ + NH_3_ plasma is a standard procedure for CMOS fabrication. But it is questionable for MKIDs on Si wafers as it could oxidize the exposed Si surface which should lead to lower *Q*_i_ values. The fact that both high-*Q*_i_ samples in Table 1 (A and C) had their resist stripped that way shows that any oxidization happening has not been significant enough to seriously increase the resonator’s dampening. But the O_2_ + N_2_ + NH_3_ plasma we used still had a significant negative impact on the detector performance: Fig. 3 shows two MKID pixels: The left one had its residual photo resist removed in the O_2_ + N_2_ + NH_3_ plasma above + 200 °C, whereas the right one had the resist removal step performed in a pure O_2_ plasma and the sample cooled at room temperature. Plotted are the frequency sweeps in the IQ plane (blue dots are measured points; blue lines are to guide the eye) as well as the detector signal without illumination in red. For the red points, the pixel’s IQ signal has been monitored for 0.1 s in darkness, resulting in 100,000 data points representing the noise cloud. As can be seen, the left sample shows significant excess noise along the direction of the frequency sweep loop (the phase direction), whereas for the sample on the right, we observe the noise to be roughly equal in both I and Q. It should be noted that the two samples had different *Q*_i_ values and microwave drive powers; the diameter of their resonator loop is therefore not identical, and thus the extent in for example the amplitude direction for the noise cannot be compared directly.

**Fig. 3:**
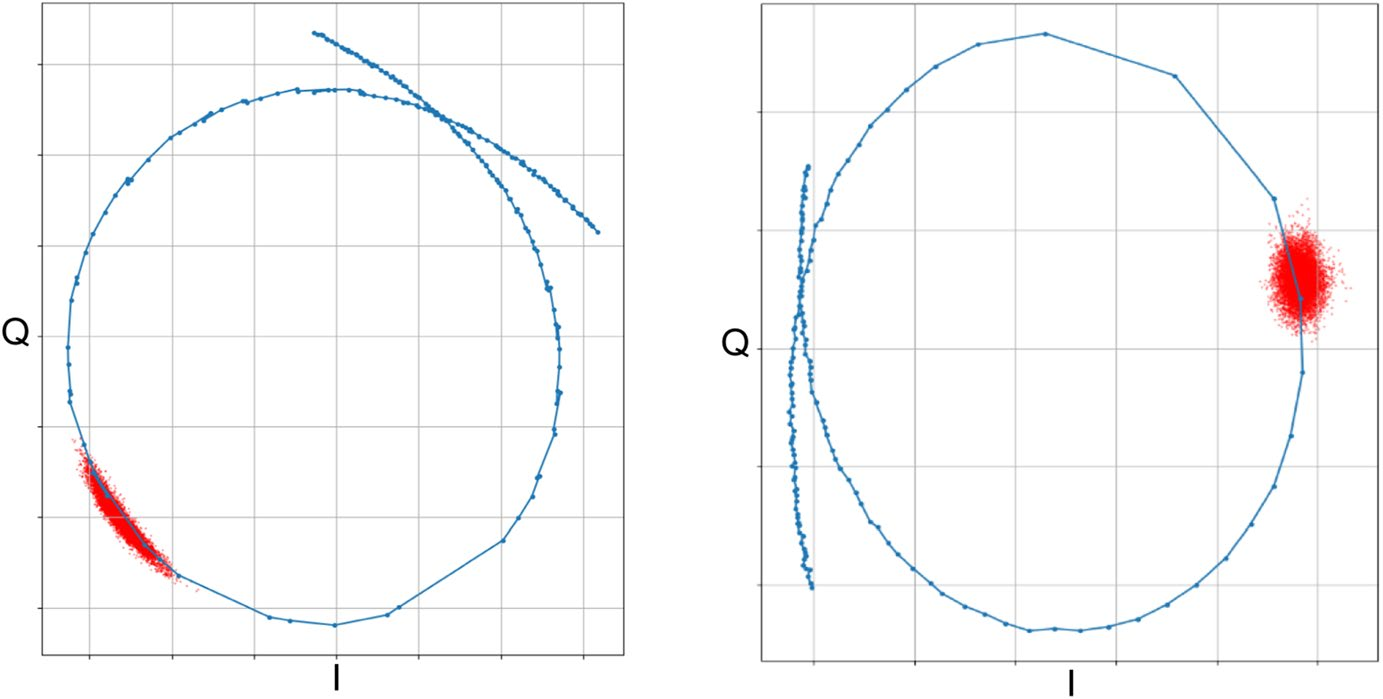
Two different TiN/Ti/TiN MKID pixels. The one on the *left* had its residual photo resist removed in an O_2_ + N_2_ + NH_3_ plasma at above +200 °C; for the one on the *right* the resist removal step has been performed in a pure O_2_ plasma and the sample cooled at room temperature. Plotted are the frequency sweeps in the IQ plane (*blue dots* are measured points; *blue lines* are to guide the eye) as well as the detector signal in darkness in *red*—for details please see text (colour figure online)

Figure 3 shows that the high-temperature resist removal step indeed had a significantly negative effect on detector performance, even though no clear degradation of *Q*_i_ has been observed. It is likely that it is the oxidization of the exposed Si surface between the capacitor fingers that leads to the observed increase in phase noise, as amorphous dielectrics are known to cause high densities of two-level system (TLS) defects [2]. The fact that we see significant phase noise caused by TLS defects but still achieve high internal quality factors could imply that our detector design is more sensitive for TLS-caused phase noise compared to TLS-caused losses, or it could indicate that we should expect even higher *Q*_i_ with further optimized sample fabrication procedures. Further work is required to get a clearer picture. Removal of the surface oxide in HF at the end of sample fabrication to test this assumption is unfortunately not possible as the Ti layer in the centre of the superconducting film gets attacked by HF too fast. After thoroughly studying the effects of annealing on our TiN/Ti/TiN multilayers, we plan to switch to sapphire substrates, in principle allowing us to use the high-temperate resist removal step again.

The best combination of critical temperature, *Q*_i_ and no-extended-phase noise we have achieved in our prototype TiN/Ti/TiN UVOIR MKIDs so far is from samples with 4 nm/25 nm/4 nm of TiN/Ti/TiN, respectively. These had a *T*_c_ of 880 mK after all lithography and fabrication steps have been performed and a typical *Q*_i_ of 60,000. As shown in Fig. 4, they performed well as single photon detectors, and we measured resolving powers of 3.1 and 2.5 for 400 and 900 nm photons, respectively—for more details please see [14]. One of the reasons the resolving power is limited is that the early prototype design used for the measurements in Fig. 4 has turned out to be too insensitive for the desired wavelength range: Even 400 nm photons that should ideally result in phase pulses of up to 180 degrees, only produced signals around 25 degrees. The sensitivity of UVOIR MKIDs for a given wavelength is mainly dictated by the superconducting bandgap (and thus *T*_c_) and the number of Cooper pairs in the inductor. Therefore, as our cryogenic system does not allow us to decrease *T*_c_ further, we have to reduce the inductor size to increase the signal strength. As further reducing the film thickness would be challenging without increasing *T*_c_, our next step is to re-design the MKID pixel itself to use smaller inductors.

**Fig. 4:**
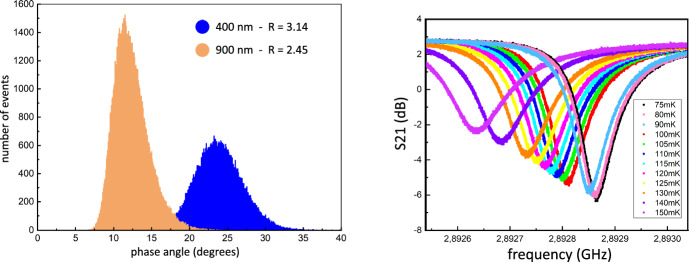
First results of a prototype TiN/Ti/TiN UVOIR MKID with a 4 nm/25 nm/4 nm structure, a *T*_c_ of 880 mK and a *Q*_i_ of 60,000. The *left* side shows the photon energy histogram for two laser lines at 400 and 900 nm. On the *right,* we plot the feedline transmission S21 for varying temperature. One issue with this early prototype is that it has been too insensitive as it only produced 25 degrees high phase pulses even for 400 nm photons (colour figure online)

The sample demonstrated in Fig. 4 had an inductor area of (ignoring the gaps) 44 × 50 µm^2^ = 2200 µm^2^. This is not significantly larger compared to inductor designs of 40 × 40 µm^2^ = 1600 µm^2^ used for TiN_x_ [7] or 44 × 30 µm^2^ = 1350 µm^2^ for PtSi [5] but turned out to be too large for TiN/Ti/TiN triple layers. With a new design using a small inductor with only 20 × 19 µm^2^ = 380 µm^2^, we hope to significantly increase single photon signal heights. As the low sensitivity we observe points towards undesired high electron densities in the film it should be promising to reduce the thickness of the Ti layer. In order to still achieve the desired T_c_ values this will necessitate using sub-stoichiometric TiN_x_ (preferably in the 2.0–3.0 K *T*_c_ range) instead of stoichiometric TiN in the multilayer instead.

In conclusion, we report our efforts on optimizing TiN/Ti/TiN triple-layer superconducting films in the *T*_c_ range around 800 mK to be used for UV/optical/near-IR MKIDs. We show that nanofabrication steps that are standard and highly tested in CMOS fabrication can still lead to unexpected and detrimental effects when used to fabricate MKIDs. We present work that is still in progress and hope to be able to provide better data and more thoroughly studied conclusions in the near future.

## References

[1] P.K. Day, H.G. LeDuc, B.A. Mazin, A. Vayonakis, J. Zmuidzinas, A broadband superconducting detector suitable for use in large arrays. Nature **425**, 817–821 (2003) 14574407 10.1038/nature02037

[2] J. Zmuidzinas, Superconducting microresonators: physics and applications. Ann. Rev. Cond. Matter Phys. **3**, 169–214 (2012) 10.1146/annurev-conmatphys-020911-125022

[3] G. Ulbricht, M. De Lucia, E. Baldwin, Applications for microwave kinetic induction detectors in advanced instrumentation. Appl. Sci. **11**(6), 2671 (2021) 10.3390/app11062671

[4] P.J. de Visser, S.A.H. de Rooij, V. Murugesan, D.J. Thoen, J.J.A. Baselmans, Phonon-Trapping-enhanced energy resolution in superconducting single-photon detectors. Phys. Rev. Appl. **16**, 034051 (2021) 10.1103/PhysRevApplied.16.034051

[5] S.R. Meeker, B.A. Mazin, A.B. Walter, P. Strader, N. Fruitwala, C. Bockstiegel, P. Szypryt, G. Ulbricht, G. Coiffard, B. Bumble, G. Cancelo, T. Zmuda, K. Treptow, N. Wilcer, G. Collura, R. Dodkins, I. Lipartito, N. Zobrist, M. Bottom, J.C. Shelton, D. Mawet, J.C. van Eyken, G. Vasisht, E. Serabyn, DARKNESS: A microwave kinetic inductance detector integral field spectrograph for high-contrast astronomy. PASP **130**, 065001 (2018) 10.1088/1538-3873/aab5e7

[6] P. Szypryt, B.A. Mazin, G. Ulbricht, B. Bumble, S.R. Meeker, C. Bockstiegel, A.B. Walter, High quality factor platinum silicide microwave kinetic inductance detectors. APL **109**, 151102 (2016) 10.1364/OE.25.025894 29041252

[7] B.A. Mazin, S.R. Meeker, M.J. Strader, P. Szypryt, D. Marsden, J.C. van Eyken, G.E. Duggan, A.B. Walter, G. Ulbricht, M. Johnson, ARCONS: A 2024 pixel optical through near-IR cryogenic imaging spectrophotometer. Publ. Astron. Soc. Pac. **125**, 1348–1361 (2013) 10.1086/674013

[8] M.R. Vissers, J. Gao, J.S. Kline, M. Sandberg, M.P. Weides, D.S. Wisbey, D.P. Pappas, Characterization and in-situ monitoring of sub-stoichiometric adjustable superconducting critical temperature titanium nitride growth. Thin Solid Films **548**, 485–488 (2013). 10.1016/j.tsf.2013.07.046 10.1016/j.tsf.2013.07.046

[9] K. Kouwenhoven, D. Fan, E. Biancalani, S.A.H. de Rooij, T. Karim, C.S. Smith, V. Murugesan, D.J. Thoen, J.J.A. Baselmans, P.J. de Visser, Resolving power of visible-to-near-infrared hybrid β−Ta/Nb−Ti−N kinetic inductance detectors. Phys. Rev. Appl. **19**, 034007 (2023). 10.1103/PhysRevApplied.19.034007 10.1103/PhysRevApplied.19.034007

[10] M.R. Vissers, J. Gao, M. Sandberg, S.M. Duff, D.S. Wisbey, K.D. Irwin, D.P. Pappas, Proximity-coupled Ti/TiN multilayers for use in kinetic inductance detectors. Appl. Phys. Let. **102**, 232603 (2013). 10.1063/1.4804286 10.1063/1.4804286

[11] A. Giachero, P.K. Day, P. Falferi, M. Faverzani, E. Ferri, C. Giordano, M. Maino, B. Margesin, R. Mezzena, R. Nizzolo, A. Nucciotti, A. Puiu, L. Zanetti, Development of microwave superconducting microresonators for neutrino mass measurement in the holmes framework. J. Low Temp. Phys. **102**, 123–130 (2016). 10.1007/s10909-015-1441-4 10.1007/s10909-015-1441-4

[12] J.E. Austermann, J.A. Beall, S.A. Bryan, B. Dober, J. Gao, G. Hilton, J. Hubmayr, P. Mauskopf, C.M. McKenney, S.M. Simon, J.N. Ullom, M.R. Vissers, G.W. Wilson, Millimeter-wave polarimeters using kinetic inductance detectors for TolTEC and beyond. J. Low Temp. Phys. **193**, 120–127 (2018). 10.1007/s10909-018-1949-5 10.1007/s10909-018-1949-5 PMC8607460 34815585

[13] H.G. Leduc, B. Bumble, P.K. Day, B. Ho Eom, J. Gao, S. Golwala, B.A. Mazin, S. McHugh, A. Merrill, D.C. Moore, O. Noroozian, A.D. Turner, J. Zmuidzinas, Titanium nitride films for ultrasensitive microresonator detectors. Appl. Phys. Lett. **97**, 102509 (2010). 10.1063/1.3480420 10.1063/1.3480420

[14] M. De Lucia, Modelling, development and characterization of microwave kinetic inductance detectors. PhD thesis, Trinity College Dublin, Ireland (2022)

